# The International Health Regulations and the imperative to strengthen global health: the case of Brazil

**DOI:** 10.1590/0037-8682-0135-2026

**Published:** 2026-07-03

**Authors:** Marcio Henrique de Oliveira Garcia, Ethel Leonor Maciel, Nisia Trindade Lima

**Affiliations:** 1 Universidade Federal de São Paulo, São Paulo, SP, Brasil.; 2 Universidade Federal do Espírito Santo, Vitória, ES, Brasil.; 3 Fundação Oswaldo Cruz, Rio de Janeiro, RJ, Brasil.

The COVID-19 pandemic exposed significant weaknesses in global health governance, not only due to insufficient regulations in the International Health Regulations (IHR) but also due to the persistent gap between the technical framework and the political commitment necessary for its full implementation. The urgent need to address these issues has prompted the 2024 amendments to the IHR, which position equity and solidarity as central tenets of global health security^1^.

In this context, Brazil serves as a strategic case study by effectively integrating global guidelines with the demands of its universal health system. The Brazilian experience demonstrates that the success of surveillance efforts relies not solely on regulations but also on a mature infrastructure that facilitates coordinated public health responses from local to federal levels. This reflects the challenges faced by Global South countries in implementing the IHR^2^.

Brazil’s recent experiences in emergency management demonstrate that health preparedness is not a static achievement but a dynamic process influenced by successive crises and regulatory changes. This institutional evolution has established operational pillars of surveillance and coordinated response, which resonate with the 2024 IHR amendments that emphasize the need for more explicit and continuous national capacities for global health security^2^. 

The effectiveness of Brazil’s responses to critical events in 2023, including zoonoses and natural disasters, underscores that overcoming institutional fragmentation relies on the ability to maintain effective communication among local, state, and federal levels. This demand for intersectoral coordination aligns directly with the 2024 IHR revisions, which aim to reduce ambiguities and delays that have historically undermined global emergency responses^2^.

Furthermore, the intersection of climate change and the maintenance of essential health services introduces an additional critical dimension to health governance. Tuberculosis exemplifies this challenge; although it may strictly qualify as a public health emergency of national or international importance, it remains a significant public health challenge in Brazil, demanding ongoing attention. The Brazilian experience shows that extreme environmental events disrupt the management of tuberculosis, revealing that the fragility of care in disaster situations serves as a sensitive indicator of the overall resilience of the health system. This scenario supports the 2024 IHR amendments’ emphasis on the need for robust national capacities to ensure structural resilience against any threats, including chronic and socially determined conditions^3^.

To strengthen its emergency response capacity, Brazil completed the Joint External Evaluation (JEE) in 2024, a process that encompasses a full cycle of monitoring and evaluating the capabilities outlined in the IHR, including the Annual Self-Assessment Report, Post-Action Reviews, and Simulation Exercises. Conducted by multi-sectoral teams, the JEE served not only as a diagnostic tool but also as a collaborative process that rigorously identified both national strengths and areas requiring strategic improvement for health security^4^ ^,^ ^5^.

The results of the JEE highlighted the strengths of Brazil’s Unified Health System, the effectiveness of the National Focal Point for the IHR, and the maturity of the National Immunization Program as key pillars of resilience. However, the report also outlined a strategic agenda requiring progress in critical areas: the institutionalization of permanent intersectoral coordination mechanisms, the strengthening of subnational capacities, and the integration of equity and gender perspectives in emergency responses. Addressing these challenges will shift the national response from a reactive framework to a sustainable and inclusive health system^4^ ^,^ ^6^.

In this context, the IHR are reaffirmed as a central legal framework, though their effectiveness hinges on addressing structural asymmetries between the Global North and South. The IHR, primarily focused on surveillance, represents only one aspect of a broader regime. Integrating the IHR with the Pandemic Agreement is essential to fill critical gaps not covered by the IHR, notably in areas such as the health industrial complex, equitable access to medical countermeasures, systems for accessing pathogens, and benefit sharing. Thus, implementing these guidelines must be viewed as an ongoing state policy that requires the coordinated integration of structures, processes, and outcomes within a long-term national strategy^5^. 

Finally, the successful implementation of this state policy requires renewed attention to subnational entities. Recent Brazilian experiences (2023-2025) demonstrate that, in a country of continental dimensions and a federative structure, the effectiveness of the IHR relies heavily on local preparedness. By strengthening its regional leadership, Brazil signals that the success of global health security depends less on centralized regulation and more on the capacity of states and municipalities, supported by the federal government, to safeguard collective health. This approach should prioritize the capillarity of surveillance actions, particularly in prevention, preparedness, and response to public health emergencies^2^ ^,^ ^6^.

## Data Availability

Not applicable.
